# Supplementary material for: Extrachromosomal DNA in the cancerous transformation of Barrett’s oesophagus
Source: Nature. 2023 Apr 12;616(7958):798–805. doi: 10.1038/s41586-023-05937-5 (PMC10132967; doi:10.1038/s41586-023-05937-5)
Supplement: Supplementary file 1 — This file contains Supplementary Methods and Supplementary Figures 1-4. [file 41586_2023_5937_MOESM1_ESM.pdf]

nature portfolio

https://doi.org/10.1038/s41586-023-05937-5

Supplementary information

Extrachromosomal DNA in the cancerous transformation of Barrett's oesophagus

In the format provided by the authors and unedited

SUPPLEMENTARY METHODS

Amplicon classification and ecDNA detection

We utilized AmpliconClassifier (AC) (version 0.4.13, available at https://github.com/jluebeck/AmpliconClassifier) to perform classification of AA outputs into different types of focal amplifications and to extract coordinates of the genomic regions corresponding to those classifications.

AC takes two primary inputs - the AA breakpoint graph file encoding genomic segment copy numbers and SV breakpoint junctions, as well as the AA cycles file encoding decompositions of the AA graph file into overlapping cyclic and/or non-cyclic paths weighted by the portion of the genomic CN they represent. AmpliconClassifier uses multiple heuristics to perform the classifications. First AC
filters the paths <10kbp, paths which significantly overlap low-complexity or repetitive regions, paths which overlap regions of the genome never exceeding CN 4.5 (not focally amplified), or which have a decomposed CN < δ (too low-frequency relative to other decompositions for reliable classification as focal amplifications). The decomposed CN (c_p) threshold, δ, for a path *p*, having a maximum genomic CN of m_p is defined as

$$\delta = \begin{cases} |p| = 1: & \min(1, \frac{m_p}{10}) \\ m_p > 7: & \min(3, \frac{m_p}{8}) \\ else: & 2.5 \end{cases}$$

For each remaining path, AC computes a length-weighted CN, called *W*, which is the product of the length of the path (in kbp) and the decomposed path's assigned copy number.

AC first assess non-filtered paths for the presence of BFB cycles using heuristics determined from manual examination of BFB-like focal amplifications in the FHCC cohort and focal amplifications in previous studies[1,2]. AC computes the fraction of breakpoint graph discordant edges which are foldback, *f*, – i.e., inverted orientation having a genomic distance < 25kbp. AC then identifies decomposed paths containing foldback junctions between segments, and using all paths computes the set of consecutive segment pairs in the paths where the two boundaries of the segments together form a foldback junction. Each segment pair is assigned its own weight equal to the decomposed copy count of the path. If the proportion of BFB-like segment pairs over all segment pairs in all paths is less than 0.295, then the amplicon is not considered to contain a BFB. Furthermore, if the total weights of pairs which are "distal" (not foldback and > 5kbp jump between endpoints) divided by the total weight of all pairs is greater than 0.5, the amplicon is not

considered to contain BFB. Lastly, if the total decomposed CN of all pairs is < 1.5, or if the total number of foldback segment pairs is < 3, or $f < 0.25$, or the decomposed CN weight of all BFB-like paths divided by the CN weight of all paths < 0.6, or the maximum genomic copy number of any region in the candidate BFB region is < 4, the amplicon is not considered to contain a BFB. If the amplicon has not failed any of these criteria, a BFB-positive status is assigned, and the BFB-like cycles (decomposed paths with a BFB foldback) are put into a set and kept separate from additional fsCNA detection inside the amplicon region.

Next, AC assess non-filtered, non-BFB paths for the presence of ecDNA cycles. If there is any cyclic path with decomposed CN > 5 and length > 100kbp, an ecDNA-positive status is assigned. If the total fraction of length-weighted CN, W, assigned to cycles exceeds 12% of the total length-weighted CN in the cycles file and more than 10kbp are inside the filtered cyclic paths, an ecDNA-positive status is assigned. Lastly, if the total length of complex cycles (cyclic paths with interior rearrangements > 5kbp) exceeds 50kbp and the region has CN > 4.5 an ecDNA-positive status is assigned. The ecDNA-like cyclic paths are then stored for subsequent analysis, including reporting of the genomic coordinates as a bed file and annotation of genes.

If the amplicon is not classified as BFB-positive and/or ecDNA-positive, and has paths consistent with focal amplification, then two other classifications are checked. If the fraction of W assigned to non-cyclic paths with rearrangements > 5kbp plus W assigned to cyclic paths is greater than 0.3 of total W in all paths, a complex non-cyclic label is assigned. If the ratio of W assigned to non-cyclic paths without rearrangements to W assigned to non-amplified paths is greater than 0.25, then the path is labeled complex non-cyclic if the breakpoint graph has > 4 discordant edges in amplified regions, otherwise a linear amplification label is assigned. If not resolved by these heuristics, the path type with the highest fraction of W is assigned.

For patient 391, for which we had 10 total biopsies or resection samples, AC classification of the chr17 BFB amplicon was not entirely consistent across all samples, despite visual inspection yielding clear evidence of BFB, likely given the high degree of structural complexity and multiple neighboring distinct focal amplifications. Three of eight focally amplified samples instead received an ecDNA classification from AC on that region despite high structural similarity across all focally amplified samples. To resolve the disagreement in focal amplification type, we applied the rule of majority to provide a single classification on those cases.

Amplicon similarity score

We compared overlapping focal amplifications to quantify amplicon similarity by quantifying the relative amounts of shared overlap in genomic coordinates and in SV breakpoint location (Supplementary Figure 1a-c). These calculations are implemented into the amplicon_similarity.py script, available in the AmpliconClassifier repository (https://github.com/jluebeck/AmpliconClassifier).

Supplementary Figure 1

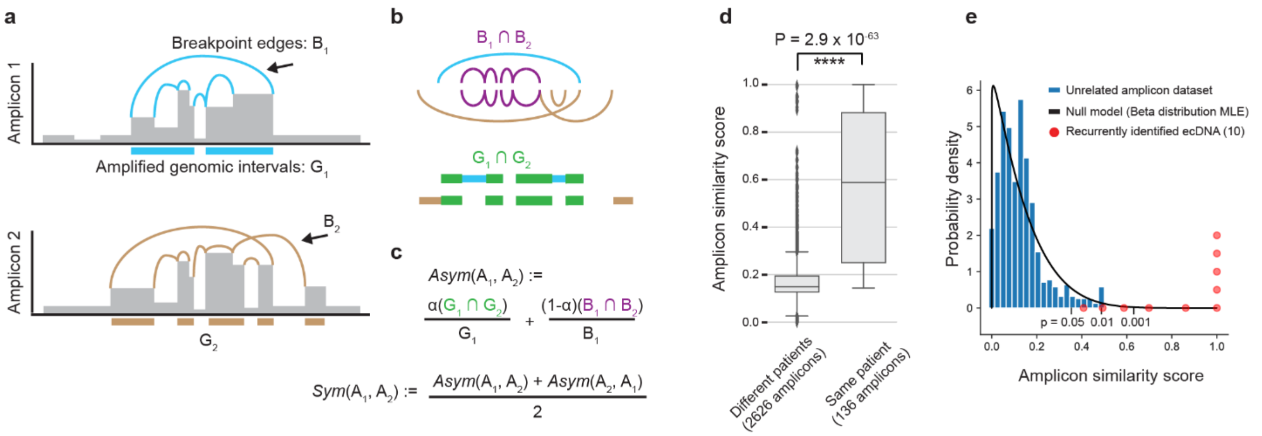

Supplementary Figure 1: Focal amplification similarity scoring.

a) Cartoon representation of two overlapping focal amplifications (A_1, A_2) consisting of a collection of genomic intervals (G_i) and breakpoints (B_i). Genomic location is shown on the x-axis and copy number on the y-axis. **b)** (Top) Representation of the relative locations of B_1, B_2 and G_1, G_2 and the resulting union of those elements, (Bottom) representation of the intersection of the elements in B_1, B_2 and G_1, G_2 highlighted in purple and green, respectively. **c)** (Top) Definition of the asymmetric similarity score function *Asym* for two overlapping amplicons. (Bottom) Definition of the symmetric similarity score, *Sym* for two overlapping amplicons, which is the average of the asymmetric scores. **d)** The distribution of maximum asymmetric similarity scores for overlapping amplicons derived from biopsies from different patients (left) and for overlapping amplicons derived from biopsies from within the same patients (right) in FHCC NCO and CO patients shows significantly higher similarity scores for amplicons derived from biopsies from within the same patients (Mann-Whitney U test). **e)** Probability density plot of amplicon similarity scores from a collection of unrelated samples with overlapping focal amplifications (blue), a beta distribution maximum-likelihood estimate of the empirical amplicon similarity score distribution (black), and

the similarity scores of overlapping ecDNA amplicons from biopsies within the same FHCC patients (red).

We defined symmetric and asymmetric amplicon similarity scores combining information from both the genomic interval overlap and the shared breakpoint junctions. An amplicon is defined as a collection of breakpoints (*B*), and genomic segments (*G*). Genomic overlap was evaluated since the number overlapping base-level coordinates in two intervals. Breakpoints were considered shared if the total distance between the two endpoints of each junction was in total measured to be less than *d* (default = 250bp). That is, for two breakpoints *x* and *y* with sorted endpoints (x_1,x_2) and (y_1,y_2), respectively, they must satisfy

$$|x_1 - y_1| + |x_2 - y_2| < d$$

The asymmetric amplicon similarity score between two amplicons A_1 and A_2 we defined as

$$Asym(A_1, A_2) = \frac{\alpha(G_1 \cap G_2)}{G_1} + \frac{(1-\alpha)(B_1 \cap B_2)}{B_1}$$

and similarly, the similarity of A_2 to A_1 is

$$Asym(A_2, A_1) = \frac{\alpha(G_2 \cap G_1)}{G_2} + \frac{(1-\alpha)(B_2 \cap B_1)}{B_2}$$

Where α is set to 0.25 by default. We then define a symmetric amplicon similarity score which is the average of the two asymmetric scores

$$Sym(A_1, A_2) = \frac{Asym(A_1, A_2) + Asym(A_2, A_1)}{2}$$

In addition to computing similarity scores for overlapping focal amplifications from the same and different patients across the three combined study groups (Cambridge, FHCC, TCGA) (Supplementary Figure 1d), we computed symmetric amplicon similarity scores for a panel of amplicons from unrelated origins derived from sequencing data published in Deshpande et al.[2], deCarvalho et al.[3], as well as Steele et al.[4] and Moody et al.[5] (using AA amplicons reported in Bergstrom et al.[6]), and the amplicons from unrelated patients in the FHCC cohort. We used the resulting distribution of 719 similarity scores for overlapping amplicons as a background null

distribution (Supplementary Figure 1e). We computed the percentile of each new amplicon similarity score in this null distribution to quantify its similarity against the panel of overlapping amplicons from unrelated origins.

We also fit a beta distribution to the empirical null symmetric similarity score distribution, using a maximum likelihood estimation approach to fit the parameters of the model. The beta distribution was selected as it provides support on the interval [0, 1], provides a higher degree of flexibility in fitting various distributions given the two shape parameters, and enables a better estimation of small p-values than the empirical dataset. We performed negative log likelihood minimization using the SciPy[7] (version 0.19.1) *fmin* function with initial parameter estimates (1.5, 10), and convergence occurred in 38 iterations.

As AmpliconArchitect may include flanking regions which are not focally amplified as part of the amplification itself, we provide a python script called feature_similarity.py, which computes the similarity scores only for given collections of focal amplification types (e.g. ecDNA), restricting the similarity score calculation only to the regions marked as ecDNA. By default, the script filters SVs which join two elements less than 2500bp away, and it redundantly filters regions that are also present in the low-complexity or low-mappability database used by AmpliconArchitect.

ISTAT analysis

We used the software ISTAT[8] (version 1.0.0) (https://github.com/shahab-sarmashghi/ISTAT) to compute the significance of the overlap of the BE & EAC driver gene regions to the predicted ecDNA regions (Supplementary Figure 2). Using the ecDNA region bed files reported by AmpliconClassifier, we took the union of these regions, and similarly extracted the gene regions associated with the BE & EAC driver gene regions using the coordinates provided by RefGene.

Supplementary Figure 2

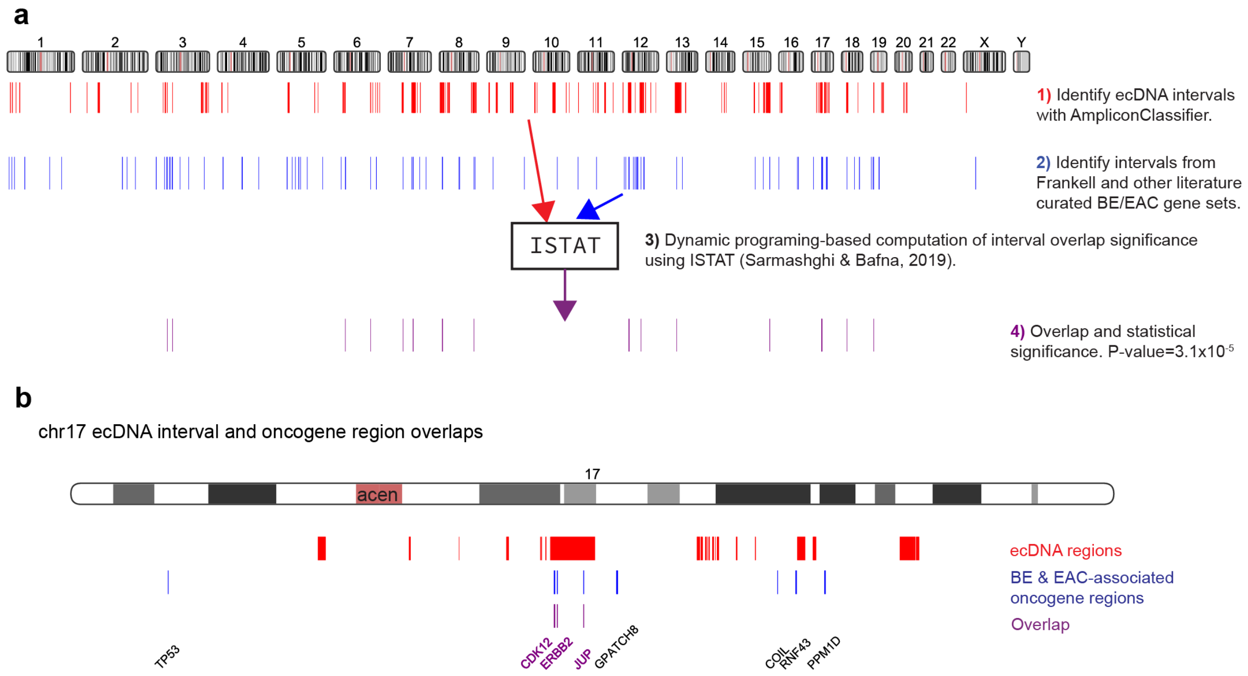

Supplementary Figure 2: ISTAT analysis.

a) ecDNA and oncogene overlap diagram showing how intervals were selected, and the methodology used to compute the overlap's statistical significance. ecDNA regions were derived from any ecDNA+ sample identified in our study. **b)** The overlap between ecDNA regions and canonical BE- and EAC-associated oncogenes for chr17.

Amplicon complexity score

AmpliconArchitect outputs a collection of (cyclic and/or non-cyclic) paths in the CN-aware breakpoint graph representing an approximate optimal balanced CN flow in the graph. As a result, non-trivial graphs may be decomposed into multiple paths, each having some copy-number assigned to the path, constrained by the total amount of CN flow available in the graph. Each path has a copy number *c*, and a length in kilobase pairs, *s*. The total length-weighted copy number of all decomposed paths we call *T*, and is given by

$$T = \sum_{i=1}^{n} s_i c_i$$

Where c_i and s_i are the copy number and length, respectively, of the *i*-th path. The values of c_i are pre-sorted in descending order for increasing *i*. For the decomposed paths of each amplicon

graph, *G*, we computed a vector representing the fraction of total CN captured by each of the *n* decompositions. We denote this sorted collection as,

$$D = \left(\frac{s_1 c_1}{T}, \ldots, \frac{s_n c_n}{T}\right)$$

We noted that there may be many low-weight CN paths, representing non- or weakly-amplified paths extracted from the graph, and thus we defined a “residual”, measured against the first percentile, *p*, (default = 80%) of weighted CN explained. We first define an index *j*, where *j* is the largest value such that

$$0 \leq j < n$$

$$\sum_{j=1}^{n} D_j < p$$

This implies that *j*+1 represents the first index such that sum of the first *j*+1 entries is equal to or exceeds *p*. The residual, ϵ, we defined as the weighted CN fractions above the first j+2 entries, is then given by

$$\epsilon = \sum_{i=j+2}^{n} D_i$$

We then defined an amplicon complexity score function *H(ϵ, D, k),* represented by the sum of entropies from the residual, the non-residual, and the total number of segments in the breakpoint graph, *k*.

$$H(\epsilon, D, k) = -\epsilon \ln \epsilon - \sum_{i=1}^{j+1} D_i \ln D_i - \ln \frac{1}{k}$$

As AmpliconArchitect amplicons may in fact encompass multiple nearby, but distinct focal amplifications, AmpliconClassifier implements this complexity score methodology with a filter such that only a subset of the values of c_i and s_i which overlap the AmpliconClassifier-detected feature are used when computing the complexity score (Supplementary Figure 3).

Supplementary Figure 3

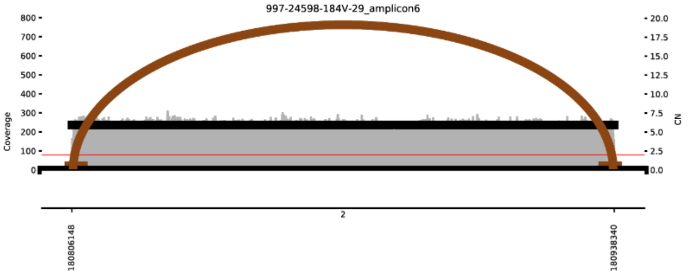

Number of CN segments in ecDNA region: 1
AA genome paths (segments and length-scaled weight):
Two paths overlapping ecDNA region:
- Path 1: cyclic, weight=0.52
- Path 2: non-cyclic, weight=0.48

Low complexity case: score 0.69

Complexity from CN segments = log(1) = 0

Complexity of non-ecDNA-like paths
= -0.48 x log(0.48) = 0.34

Complexity of ecDNA-like paths
= -0.52 x log(0.52) = 0.35

Total complexity score:
= complexity from number of segments +
ecDNA complexity + residual complexity +
= 0 + 0.34 + 0.35
= 0.69

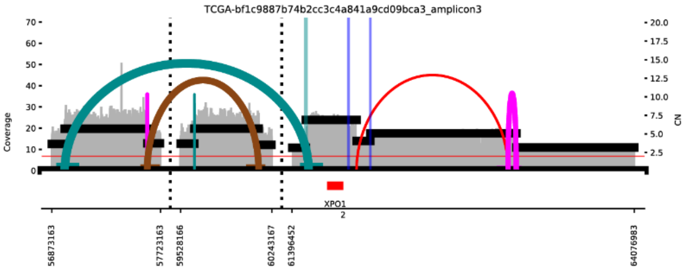

Number of CN segments in ecDNA region: 10
AA genome paths (segments and length-scaled weight):
Eight paths overlapping ecDNA regions:
- Two cyclic paths, both in top 80% of weights: 0.21, 0.07
- Six non-cyclic paths, combined weight = 0.72

Medium complexity case: score 3.05

Complexity from CN segments = log(10) = 2.30

Complexity of non-ecDNA-like paths
= -0.72 x log(0.72) = 0.24

Complexity of ecDNA-like paths
= -0.21 x log(0.21) + -0.07 x log(0.07) = 0.51

Total complexity score:
= 2.30 + 0.24 + 0.51
= 3.05

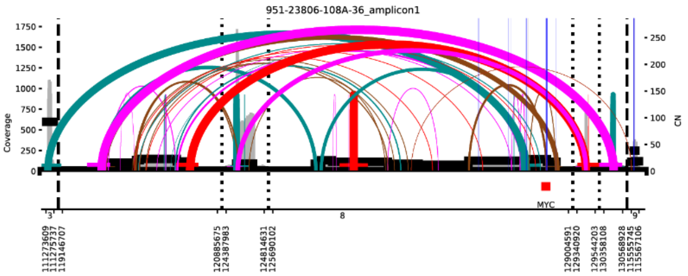

Number of CN segments in ecDNA region: 84
AA genome paths (segments and length-scaled weight):
Twenty-four paths overlapping ecDNA regions:
- Eleven cyclic paths in top 80% of all weights:
D = [0.23, 0.14, 0.077, ... , 0.023, 0.014, 0.012]
- Thirteen non-cyclic paths & cyclic paths in bottom 20% of weights, combined weight = 0.30

High complexity case: score 6.44

Complexity from CN segments = log(84) = 4.43

Complexity of non-ecDNA-like paths
= -0.30 x log(0.30) = 0.36

Complexity of ecDNA-like paths
= $-\sum D_i \times \log(D_i)$ = 1.65

Total complexity score:
= 4.43 + 0.36 + 1.65
= 6.44

Supplementary Figure 3: Focal amplification complexity score analysis.
Three worked examples of the ecDNA complexity calculation, demonstrating increasing structural complexity scores with increasing numbers of genomic paths and copy-number states.

Association of ecDNA status to purity, cellularity, and ploidy
We found no significant relationships between ecDNA status and estimated purity for the Cambridge EAC or FHCC CO study samples (Mann-Whitney U test, p-values = 0.93, 0.12, respectively, Supplementary Figure 4a-b). With histology-based cellularity estimates available for the Cambridge study samples, neither did we find a relationship between tumor cellularity and WGS-based ecDNA status (Mann-Whitney U test p-value=0.057, Supplementary Figure 4c). Lastly, we found no significant relationship between ecDNA status with WGS sequence coverage (Mann-Whitney U test, p-value=0.96, Supplementary Figure 4d), suggesting that sample-specific properties did not significantly influence the discovery of ecDNA with these samples.

However, when we examined estimated ploidy in relation to ecDNA status, we found significantly higher ploidy in ecDNA+ samples (Mann-Whitney U test, p-values=2.3×10^{-4} and 0.021 for Cambridge and FHCC respectively, Supplementary Figure 4e-f), suggesting a link between ecDNA and genomic instability.

Supplementary Figure 4

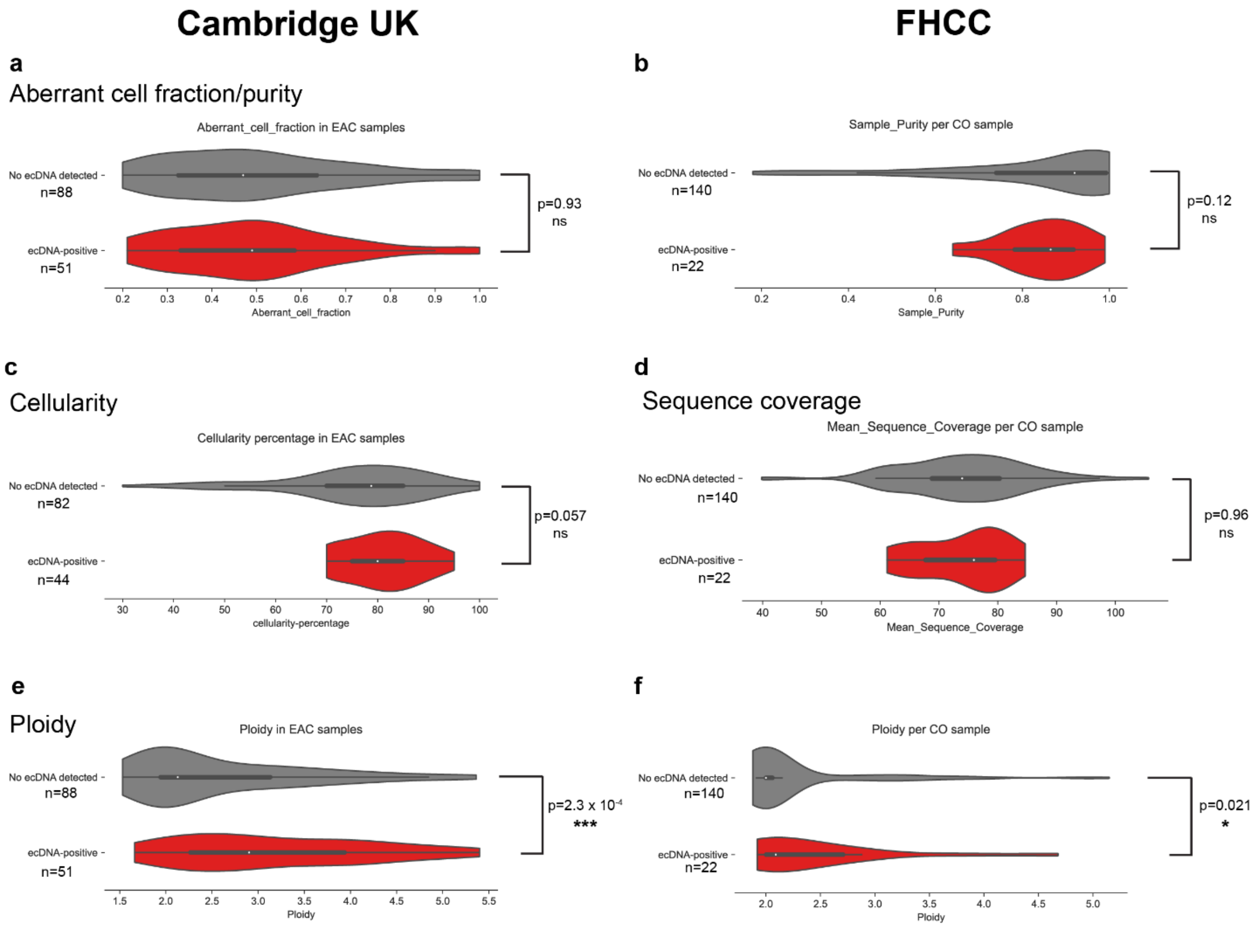

Supplementary Figure 4: ecDNA and sample properties.

Left and right columns provide measurements of sample properties versus ecDNA status for Cambridge EAC and FHCC CO study samples, respectively. **a)** Aberrant cell fraction estimate (ASCAT-based) for Cambridge EAC WGS samples. **b)** Purity estimate (pASCAT-based) for FHCC CO WGS samples. **c)** Histological cellularity distribution for Cambridge EAC histology samples. **d)** Sequencing coverage distribution for FHCC CO WGS samples. **e)** Ploidy estimate (ASCAT-based) for Cambridge EAC WGS samples. **f)** Ploidy estimate (pASCAT-based) for FHCC CO WGS samples.

Supplementary Information references

1. Kim, H. *et al.* Extrachromosomal DNA is associated with oncogene amplification and poor outcome across multiple cancers. *Nat. Genet.* **52**, 891–897 (2020).
2. Deshpande, V. *et al.* Exploring the landscape of focal amplifications in cancer using AmpliconArchitect. *Nat. Commun.* **10**, 1–14 (2019).
3. Decarvalho, A. C. *et al.* Discordant inheritance of chromosomal and extrachromosomal

DNA elements contributes to dynamic disease evolution in glioblastoma. *Nat. Genet.* **50**, 708–717 (2018).

4. Steele, C. D. *et al.* Undifferentiated Sarcomas Develop through Distinct Evolutionary Pathways. *Cancer Cell* **35**, 441-456.e8 (2019).
5. Moody, S. *et al.* Mutational signatures in esophageal squamous cell carcinoma from eight countries with varying incidence. *Nat. Genet.* **53**, 1553–1563 (2021).
6. Bergstrom, E. N. *et al.* Mapping clustered mutations in cancer reveals APOBEC3 mutagenesis of ecDNA. *Nature* **602**, 510–517 (2022).
7. Virtanen, P. *et al.* SciPy 1.0: fundamental algorithms for scientific computing in Python. *Nat. Methods* **17**, 261–272 (2020).
8. Sarmashghi, S. & Bafna, V. Computing the statistical significance of overlap between genome annotations with iStat. *Cell Syst.* **8**, 523 (2019).
