# Supplementary material for: Extrachromosomal DNA in the cancerous transformation of Barrett’s oesophagus
Source: Nature. 2023 Apr 12;616(7958):798–805. doi: 10.1038/s41586-023-05937-5 (PMC10132967; doi:10.1038/s41586-023-05937-5)
Supplement: Supplementary file 2 — Reporting Summary [file 41586_2023_5937_MOESM2_ESM.pdf]

nature portfolio

Corresponding author(s): Paul S. Mischel

Last updated by author(s): 2/10/2023

Reporting Summary

Nature Portfolio wishes to improve the reproducibility of the work that we publish. This form provides structure for consistency and transparency in reporting. For further information on Nature Portfolio policies, see our Editorial Policies and the Editorial Policy Checklist.

Statistics

For all statistical analyses, confirm that the following items are present in the figure legend, table legend, main text, or Methods section.

n/a	Confirmed	
☐	☒	The exact sample size (*n*) for each experimental group/condition, given as a discrete number and unit of measurement
☐	☒	A statement on whether measurements were taken from distinct samples or whether the same sample was measured repeatedly
☐	☒	The statistical test(s) used AND whether they are one- or two-sided<br>*Only common tests should be described solely by name; describe more complex techniques in the Methods section.*
☐	☒	A description of all covariates tested
☒	☐	A description of any assumptions or corrections, such as tests of normality and adjustment for multiple comparisons
☐	☒	A full description of the statistical parameters including central tendency (e.g. means) or other basic estimates (e.g. regression coefficient) AND variation (e.g. standard deviation) or associated estimates of uncertainty (e.g. confidence intervals)
☐	☒	For null hypothesis testing, the test statistic (e.g. *F*, *t*, *r*) with confidence intervals, effect sizes, degrees of freedom and *P* value noted<br>*Give P values as exact values whenever suitable.*
☒	☐	For Bayesian analysis, information on the choice of priors and Markov chain Monte Carlo settings
☒	☐	For hierarchical and complex designs, identification of the appropriate level for tests and full reporting of outcomes
☒	☐	Estimates of effect sizes (e.g. Cohen's *d*, Pearson's *r*), indicating how they were calculated

Our web collection on statistics for biologists contains articles on many of the points above.

Software and code

Policy information about availability of computer code

Data collection	No data were generated for this study. All data can be downloaded from the included repositories, links and references. The BE and HGD Cambridge UK cohort whole-genome sequencing data, histology and metadata was previously published in Katz-Summercorn et al.13 and whole-genome sequencing data are available through the European Genome-phenome Archive (EGA) under accession ID EGAD00001006349. The EAC Cambridge UK cohort whole-genome sequencing data, histology and metadata were downloaded from the International Cancer Genome Consortium (ICGC) at https://dcc.icgc.org/. The FHCC cohort whole genome sequencing samples, histology and metadata were previously published in Paulson et al.14 and whole-genome sequencing data are available from the NCBI dbGaP database under accession ID phs001912.v1.p1. All sequencing data, histology and metadata for TCGA were downloaded from the GDC (https://gdc.cancer.gov/) under accession ID phs000178.v11.p8. We have uploaded the AmpliconArchitect and AmpliconClassifier output files to FigShare at https://doi.org/10.6084/m9.figshare.21893826.
Data analysis	AmpliconSuite-pipeline (0.1203.12) https://github.com/jluebeck/AmpliconSuite-pipeline<br>AmpliconArchitect (1.2) (https://github.com/jluebeck/AmpliconArchitect)<br>AmpliconClassifier (0.4.13) (https://github.com/jluebeck/AmpliconClassifier)<br>CycleViz (0.1.1) (https://github.com/jluebeck/CycleViz)<br>SciPy (1.9.1)<br>ASCAT (v2.3)<br>CNVKit (version 0.9.7 and version 0.9.6)<br>ISTAT (1.0.0)<br>BWA-mem (version 0.7.17 and version 0.6.2-r126)<br>GATK IndelRealigner (3.4-0-g7e26428)<br>SNPeff (4.2)

Strelka (2.0.15)
Variant Effect Predictor (78)

For manuscripts utilizing custom algorithms or software that are central to the research but not yet described in published literature, software must be made available to editors and reviewers. We strongly encourage code deposition in a community repository (e.g. GitHub). See the Nature Portfolio guidelines for submitting code & software for further information.

Data

Policy information about availability of data

All manuscripts must include a data availability statement. This statement should provide the following information, where applicable:

- Accession codes, unique identifiers, or web links for publicly available datasets
- A description of any restrictions on data availability
- For clinical datasets or third party data, please ensure that the statement adheres to our policy

The BE and HGD Cambridge UK cohort whole-genome sequencing data, histology and metadata was previously published in Katz-Summercorn et al.13 and whole-genome sequencing data are available through the European Genome-phenome Archive (EGA) under accession ID EGAD00001006349. The EAC Cambridge UK cohort whole-genome sequencing data, histology and metadata were downloaded from the International Cancer Genome Consortium (ICGC) at https://dcc.icgc.org/. The FHCC cohort whole genome sequencing samples, histology and metadata were previously published in Paulson et al.14 and whole-genome sequencing data are available from the NCBI dbGaP database under accession ID phs001912.v1.p1. All sequencing data, histology and metadata for TCGA were downloaded from the GDC (https://gdc.cancer.gov/) under accession ID phs000178.v11.p8. We have uploaded the AmpliconArchitect and AmpliconClassifier output files to FigShare at https://doi.org/10.6084/m9.figshare.21893826.

Human research participants

Policy information about studies involving human research participants and Sex and Gender in Research.

Reporting on sex and gender	Sex and gender data was not collected in this study, as we reanalyzed previously published collections of samples from human research participants. In all study sources, sex and gender were previously reported and analyzed.
Population characteristics	Population characteristics for each study used as a source of data are described in<br>FHCC cohort: Paulson et al., Nature Communications 2022<br>Cambridge Cohort: Katz-Summercorn et al., Nature Communications 2022 and The ICGC/TCGA Pan-Cancer Analysis of Whole Genomes Consortium, Nature 2020.<br>TCGA: The Cancer Genome Atlas Research Network, Nature 2017
Recruitment	Recruitment for each study used as a source of data are described in<br>FHCC cohort: Paulson et al., Nature Communications 2022<br>Cambridge Cohort: Katz-Summercorn et al., Nature Communications 2022 and The ICGC/TCGA Pan-Cancer Analysis of Whole Genomes Consortium, Nature 2020.<br>TCGA: The Cancer Genome Atlas Research Network, Nature 2017
Ethics oversight	Ethics oversight for each study used as a source of data are described in<br>FHCC cohort: Paulson et al., Nature Communications 2022<br>Cambridge Cohort: Katz-Summercorn et al., Nature Communications 2022 and The ICGC/TCGA Pan-Cancer Analysis of Whole Genomes Consortium, Nature 2020.<br>TCGA: The Cancer Genome Atlas Research Network, Nature 2017

Note that full information on the approval of the study protocol must also be provided in the manuscript.

Field-specific reporting

Please select the one below that is the best fit for your research. If you are not sure, read the appropriate sections before making your selection.

☒ Life sciences ☐ Behavioural & social sciences ☐ Ecological, evolutionary & environmental sciences

For a reference copy of the document with all sections, see nature.com/documents/nr-reporting-summary-flat.pdf

Life sciences study design

All studies must disclose on these points even when the disclosure is negative.

Sample size	For the Cambridge UK cohort, no sample size calculation was performed. The sample size was made as large as it could be possible based on the availability of suitable material for sequencing. For the Fred Hutch cohort, the 80 patients with Barrett's esophagus (40 with cancer outcome and 40 without cancer outcome) were selected from a previously published case-cohort study of 248 patients from Li et al, Cancer Prev Res, 2014 (PMID 24253313), in which somatic chromosomal alterations (SCA) had been characterized every two centimeters (cm) in the Barrett's segment. All samples used in this research were published in DOI 10.1038/s41467-022-29767-7 and 10.1038/s41467-022-28237-4
Data exclusions	No data was excluded as it is a re-analysis of previous published whole-genome sequencing data.

Replication	This is a re-analysis of whole-genome sequencing data. Replication is not applicable.
Randomization	For the Cambridge UK cohort, randomization is not applicable, because samples were grouped based on the grade of pathology of the patients. For the Fred Hutch cohort, for each cancer outcome case, non-cancer outcome controls were randomly matched on baseline total SCA, age at T1, time between T1 and T2, and gender for the comparisons of the two groups.
Blinding	All individuals performing whole-genome sequencing analysis were blinded to cancer outcome.

Reporting for specific materials, systems and methods

We require information from authors about some types of materials, experimental systems and methods used in many studies. Here, indicate whether each material, system or method listed is relevant to your study. If you are not sure if a list item applies to your research, read the appropriate section before selecting a response.

Materials & experimental systems

n/a	Involved in the study
☒	☐ Antibodies
☒	☐ Eukaryotic cell lines
☒	☐ Palaeontology and archaeology
☒	☐ Animals and other organisms
☒	☐ Clinical data
☒	☐ Dual use research of concern

Methods

n/a	Involved in the study
☒	☐ ChIP-seq
☒	☐ Flow cytometry
☒	☐ MRI-based neuroimaging
